# Malaria: moving beyond the search for magic bullets

**DOI:** 10.15252/emmm.202318727

**Published:** 2023-10-04

**Authors:** Elena Levashina, Dominique Soldati‐Favre, Andrew P Waters, Friedrich Frischknecht, Julian C Rayner

**Affiliations:** ^1^ Vector Biology Unit Max Planck Institute for Infection Biology Berlin Germany; ^2^ Department of Microbiology and Molecular Medicine, Faculty of Medicine University of Geneva Geneva Switzerland; ^3^ Wellcome Centre for Integrative Parasitology, Institute of Infection, Immunity, and Inflammation University of Glasgow Glasgow UK; ^4^ Integrative Parasitology, Center for Infectious Diseases Heidelberg University Medical School Heidelberg Germany; ^5^ German Center for Infection Research, partner site Heidelberg Heidelberg Germany; ^6^ Cambridge Institute for Medical Research University of Cambridge Cambridge UK

**Keywords:** Microbiology, Virology & Host Pathogen Interaction

## Abstract

Round table discussion on challenges and opportunities in malaria research with Elena Levashina, Dominique Soldati‐Favre, Andrew Waters, Friedrich Frischknecht, and Julian Rayner.

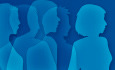

Malaria is a devastating infectious disease that caused an estimated 200 million deaths in the 20^th^ century alone and continues to infect over 200 million people every year, leading to the loss of over half a million lives, mostly those of young children. The *Plasmodium* spp. parasites that cause malaria and the mosquito vectors that transmit them were both identified in the last two decades of the 19^th^ century. Most cases and fatalities are due to infections with *Plasmodium falciparum*, but other species are also important causes of disease with *Plasmodium vivax* being the most widely geographically distributed. Methods to treat malaria long predate our understanding of its cause, with extracts of the cinchona tree bark and other natural remedies being used to treat “swamp fever” for centuries. As research moved into the modern age, control efforts became ever more sophisticated, with insecticides and synthetic antimalarial drugs significantly reshaping the malaria world map and helping eradicate the disease from locations where it was endemic such as Europe and North America. However, while progress against malaria has undoubtedly been made in many countries, the emergence and spread of both antimalarial drug resistance and insecticide resistance have led to cycles of progress and reversal, and *Plasmodium* parasites stubbornly remain a major global health challenge and cause of infant mortality in many subtropical regions with the highest burden in Sub‐Saharan Africa.

Over the past century, scientific research has revolutionized our understanding of the life cycle of *Plasmodium* parasites, their interactions with the mosquito and the human (or vertebrate) hosts and revealed new drug, vaccine, and insecticide candidates that have the potential to contribute to malaria control. While many drugs and insecticides have been widely applied, one control measure the field still lacks however is a widely used and effective vaccine. Sequencing the genome of the SARS‐CoV2 virus immediately revealed a single clear vaccine target, a conserved protein known to be important for invasion of other related viruses, and kickstarted subsequent vaccine development that saved millions of lives. By contrast, the genome of *P. falciparum*, completely sequenced more than two decades ago, contains 5,000 genes and it remains an enormous challenge to identify the best vaccine target(s). This picture may however be about to change. The first malaria vaccine (RTS,S or Mosquirix) was approved by WHO in 2021 and is now being rolled out in some African countries, while another closely related vaccine (R21) recently became only the second to undergo Phase III large‐scale testing, with results eagerly anticipated and much‐trailed. Both are based on the predominant surface protein that coats the sporozoite, the stage of the parasite that is transmitted by the mosquito to humans. Following transmission, sporozoites make their way from the site of the mosquito bite to the liver, where they invade and develop inside hepatocytes. Vaccine elicited antibodies and T‐cells are thought to block the migration of parasites in the skin and attack the parasites as they develop in the liver. While delivering the first approved malaria vaccine was a massive achievement based on decades of work, trials with RTS,S only showed a vaccine efficacy of 30–50% after four injections with immunity declining after a year, signaling that much more development is needed. R21 could provide another step change, but the history of malaria vaccines has been long and complex.

During the XIX BioMalPar conference at EMBL, Heidelberg, an annual gathering of malaria researchers from around the world, *EMBO Molecular Medicine* scientific editor Zeljko Durdevic hosted a round table discussion of EMBO Members Andrew Waters, Dominique Soldati‐Favre, Elena Levashina, Friedrich Frischknecht and Julian Rayner to discuss the promise of these vaccines, how research has contributed to understanding of malaria biology and the development of control measures, and give their view of the challenges and opportunities ahead for the field.

**Julian Rayner (JR):** We are having this discussion as we await the results of a Phase III trial for the R21 vaccine, that are widely believed to be encouraging. However, the history of malaria control has been marked by a recurring pattern where initial excitement is followed by problems that emerge over time. This is particularly noticeable in the case of both anti‐malarial drugs and insecticides, whereby new interventions are introduced only to be blunted and sometimes lost entirely by the evolution of resistance. Similar concerns exist about the evolution of vaccine escape, and earlier trials do provide some evidence that it could be a concern, even for these latest generations of vaccines.

**Friedrich Frischknecht (FF):** Indeed, there is data indicating that when looking at the locations where the RTS,S vaccine was initially tested, vaccine protection depended in part on the infecting parasites' genotype for the CSP (circumsporozoite protein) on which the vaccine was based. This suggests that adaptation dynamics could lead to the emergence of parasites that become resistant to the blocking effects of antibodies or T cell responses induced by a vaccine. It is plausible that mRNA vaccines could offer a solution, as they possess the advantage of rapid production in diverse formats, eliminating the need for complex processes such as viral vectors or vaccines based on expressed proteins, and enabling more rapid generation of new vaccines to keep up with the emergence of new variants. However, whether these new mRNA vaccines will be able to generate the needed levels of antibodies, which are likely to be much higher than for SARS‐CoV2, and strength of T‐cell responses is not yet known.

**Elena Levashina (EL):** We are discussing the potential of a new generation malaria vaccine, which essentially involves expression of potent anti‐CSP B cell epitopes on nanoparticles along with the use of another adjuvant. Although it aims to elicit stronger immune responses, we must be precise and not overly raise hopes of groundbreaking advancements. Scientists are actively researching the best epitopes to target, particularly in the context of transmission‐blocking vaccines, whether mRNA‐based or protein‐based. However, there is still much to learn about the specific functions and regulation of these epitopes (proteins). It is a complex puzzle requiring sustained efforts to unravel the molecular details of parasite biology, similar to what we have achieved with antiviral vaccines.

**JR:** I think you (EL) have identified the core challenge that the field faces. There are over 5,000 genes in the malaria genome and for most of them the function is unknown. Compounding complexity is the extensive natural diversity observed within the parasite genome across the global distribution of malaria parasites. Rather than a single *P. falciparum* genome, there exist millions of *P. falciparum* genomes, and this variation could potentially impact vaccine efficacy. In addition, as David Conway highlighted years ago, the vaccines that have progressed the furthest in development are actually those based on *P. falciparum* genes that were cloned and sequenced first. This is due to the extensive study and familiarity surrounding those genes. It is quite possible that there are superior targets waiting to be discovered, but we just have not got to them yet, because it is such a large problem.

**Andrew Waters (AW):** As a community, we have also yet to definitively identify the correlates of protection, which means we lack a clear benchmark to evaluate whether a vaccine is generating the desired immune responses. Consequently, administering a vaccine to an individual and expecting it to provide protection remains somewhat speculative. On a parallel track, one alternative is to adopt a whole organism approach, as several initiatives have pursued. While this approach has the advantage that it simultaneously presents numerous epitopes and could yield more favorable outcomes, it does not circumvent the challenge Julian (JR) highlighted—the presence of millions of genomes shaped by evolutionary and immune pressures, not to mention the diverse genotype, phenotype, and protein display resulting from recent drug pressures. This raises questions about the feasibility of a single universally administered vaccine. Perhaps tailoring vaccines based on specific requirements is necessary. One example of such an approach targets receptors that are predominantly expressed on the surface of the parasite‐infected erythrocyte (PIE) that recognize chondroitin sulphate A (CSA). This ligand is exclusively expressed on the placenta during pregnancy and PIEs expressing the CSA receptor (VAR2CSA) therefore sequester exclusively to the placenta. The architecture of the placenta facilitates the establishment of a cryptic cycle in the environs of the placenta leading to localized parasite multiplication, increasing obstruction of placental function with potentially fatal outcome for mother and unborn child. This is pregnancy‐associated malaria but vaccines are under development that target VAR2CSA that is largely responsible for mediating sequestration with a view to preventing PAM in the susceptible primigravid mothers.

**EL:** Another important aspect are our available laboratory tools that are predominantly limited to one working *Plasmodium falciparum* line, the NF54 strain, which was isolated decades ago before drug resistance became widespread. This limitation becomes even more pronounced when attempting to study mosquitoes. In mosquito biology, we primarily work with a small number of strains, often of unknown origin and confined to laboratory conditions for several decades. This restriction hampers our ability to address genetic diversity, adaptation, and develop meaningful models. Our current tools therefore fail to adequately represent the complexity observed in the field. We are aware that parasite lines exhibit significant variations in behavior, growth rates, and other characteristics. The variability among mosquitoes is also substantial, yet often overlooked in our studies. Furthermore, cultivating human *Plasmodium* parasites in a complete life cycle with all their stages is exceptionally challenging.

**Dominique Soldati‐Favre (DSF):** When we examine the historical trajectory of vaccine development against intricate eukaryotic infectious diseases like malaria, the record of success is scarce. Even if we contemplate HIV, which could be considered as a manageable challenge, achieving efficacious vaccine has proven to be difficult, to say the least. I think it would be overly optimistic to envision that a monovalent or even multivalent vaccine, whether protein‐based or RNA‐based, will provide a straightforward solution. These parasites possess exceedingly sophisticated strategies to evade the immune system, rendering our efforts to counteract them challenging. That is why I am cautious about the extent of enthusiasm we should place on vaccination.

**JR:** One might consider the saying “do not let the perfect be the enemy of the good.” These new approaches, despite potential concerns and risks of escape, could still save lives and buy valuable time. It resembles the drug development field's experience over the past two decades, with new drugs emerging in successive waves while resistance evolves. Nonetheless, these drugs remain useful and save lives until they can no longer be effective. The challenge lies in maintaining a continuous pipeline of new tools to replace the old ones. Perhaps I am overly optimistic, but in the realm of drug development, there seems to be a more promising landscape now compared to 20 years ago.

**FF:** I agree, there is room for optimism as research is speeding up thanks to the many technical developments and discoveries over the past decades, many of which were first presented at the BioMalPar meeting, and which now enable translational approaches. For example, Dominique (DSF) made a remarkable discovery—plasmepsin inhibitors that block egress of the parasite from the red blood cell. We just heard a captivating talk by Leann Tilley, who began with fundamental cell biology and biochemical experiments and has now generated potential drug candidates. And that's just two examples. It appears that scientists who persist in their research for several decades often uncover such candidates, and hopefully, a few of them will eventually succeed to apply them and save lives.

**DSF:** When it comes to the drug discovery pipeline, there has been a deliberate shift toward conducting phenotypic screening of millions of compounds, leading to a promising array of new chemical entities and inhibitors. The availability of a range of experimental tools to identify the target, which was previously challenging, has greatly contributed to this progress. Additionally, the optimization process has become faster with the aid of modeling and artificial intelligence. Thus, the future appears more promising. However, we must acknowledge the perpetual catch‐22 situation. We need to continuously push forward, exploring new candidates and strategies without any room for complacency. Perseverance is the key.

**FF:** Perhaps there is a crucial point to convey here. The complexity of the malaria parasite is such that we are left without a singular solution. There may never be a “magic bullet”, as was once hoped when chloroquine and DDT (Dichlorodiphenyltrichloroethane) emerged in the 1950s. Although these were highly effective molecules against the parasite and insects, they fell short of completely eradicating the parasite worldwide. However, we did achieve a tremendous success in eliminating it from Europe and North America. This achievement should not be underestimated and also not undermined.

**EL:** Before the discovery of DDT, the early insecticides used in the first campaign to eradicate malaria in Italy were highly toxic and unimaginable for present‐day use. As we develop insecticides, we must consider ethical boundaries and avoid using poisons to solve one problem while creating another. However, there is still much we do not understand about the relationship between insecticides and resistance in insects. The selection of pre‐existing mutations rather than the emergence of new ones contributes to their rapid spread. This issue is similar to antibiotic resistance in bacteria. Without a deeper understanding of this mimicry, developing resistant‐proof insecticides becomes challenging.

Gene drive technology is an additional tool for controlling insect populations, offering precise and efficient control over specific genotypes. However, targeting specific mosquito species in the field presents challenges, as natural mosquito populations are complex. Selecting the right species and ensuring accurate targeting are significant concerns. Additionally, the lack of tools for studying certain mosquito species in laboratories complicates the process. It raises questions about potential unintended consequences and the ecological impact of removing or suppressing particular species.

**JR:** As Freddy (FF) mentioned earlier, there is no “magic bullet”, a term first coined by Paul Ehrlich in the early 1900s, and the same may well apply to gene drives. Although gene drive technology has not been extensively tested, we should anticipate that it, like drugs and vaccines, could also select for resistance. While it is an exciting and promising tool with many possibilities, as scientists we simply have to be cautious about presenting it as the perfect and single solution.

**FF:** The use of Wolbachia‐infected mosquitoes to control the spread of diseases like dengue and yellow fever is a good example. While they have shown success in certain settings, their effectiveness varies, and they are not a guaranteed solution to eliminate these diseases entirely. They are a valuable tool in public health efforts, but not a “magic bullet”. Similarly, even with a highly effective vaccine like the measles vaccine, there are social and other factors that can hinder its distribution and result in ongoing measles cases. Again, making the case that control of malaria needs multiple approaches.

**AW:** It is important to communicate this soberly and ensure that the popular press conveys the correct level of expectation to the wider public regarding any breakthroughs. We must remain vigilant, continue developing new compounds and approaches, and consider reusing existing options. With a large arsenal of drugs, responsive governments, and ongoing research, there is reason to be optimistic. If we are allowed to progress and generate more therapeutic molecules, the situation can only improve—it should improve.

**JR:** Indeed, it should improve. I am constantly amazed by which speed new tools are developed. When I reflect on my own experience starting to work in this field 20 years ago, the progress we have made in understanding the parasite, its diversity, and our ability to manipulate it, as well as the diversity of mosquito vectors, to knockdown genes in the vector and our knowledge of immune responses, is absolutely incredible. The depth of information we now have is highly encouraging and signifies the need to continue our long‐term efforts. However, it is crucial to expedite communication and coordination between fundamental biology, policy, and implementation.

**DSF:** I think the faster progress is not only due to technological advancements, but also because the new generation of scientists embraces interdisciplinary approaches without fear. There is a willingness to bridge the gap between fundamental observations and translational products. Even within academia, there is increased exposure to translational research, unlike the siloed approach of the past. This shift presents tremendous potential for innovation and collaboration.

**AW:** Also, the scientific community is moving toward early data sharing, recognizing the value it holds for others to analyze and extract insights. This collaborative approach plays a significant role in expediting research. Credit must be given to charities and funding agencies that have actively encouraged and enforced this practice.

**DSF:** Indeed, data sharing and open science is a revolution, and it has contributed to our increased speed in research. I think journals play a significant role in facilitating this process. Data sharing has become more immediate through platforms like preprint servers and bioRxiv, allowing for corrections and widespread access. While maintaining trustable peer review is important, the focus should be on preserving high‐quality data sharing to ensure its usefulness to all.

**EL:** I completely agree. Collaboration and open communication are crucial for our success. The rapid progress in drug development will inevitably pave the way for faster and improved vaccine development and vice versa. It opens new possibilities and fields of exploration. This positive development in one aspect will likely catalyze advancements in other areas as well.

**FF:** That links all back to our optimistic viewpoint about what can be achieved without losing sight of the suffering by the most affected who urgently need help and the challenges this entails.

**JR:** It is important to communicate that while announcements about a new vaccine or drug may be very exciting, it is essential to recognize that they do not signify the end of the journey. The risk lies in perceiving them as a magic bullet, leading to potential withdrawal of support from funders and a misguided belief that the problem is solved. There are numerous reasons to be optimistic, as you all mentioned, but we must maintain our momentum and drive our progress.

## Reading list

Beeson JG, Kurtovic L, Valim C, Asante KP, Boyle MJ, Mathanga D, Dobano C, Moncunill G (2022) The RTS,S malaria vaccine: current impact and foundation for the future. *Sci Transl Med* 14: eabo6646

Cowman AF, Healer J, Marapana D, Marsh K (2016) Malaria: biology and disease. Cell 167: 610–624

Datoo MS, Natama MH, Some A, Traoré O, Rouamba T, Bellamy D, Yameogo P, Valia D, Tegneri M, Ouedraogo F *et al* (2021) Efficacy of a low‐dose candidate malaria vaccine, R21 in adjuvant Matrix‐M, with seasonal administration to children in Burkina Faso: a randomised controlled trial. *Lancet* 397: 1809–1818

Draper SJ, Sack BK, King CR, Nielsen CM, Rayner JC, Higgins MK, Long CA, Seder RA (2018) Malaria vaccines: recent advances and new horizons. *Cell Host Microbe* 24: 43–56

Gamain B, Chêne A, Viebig NK, Tuikue Ndam N, Nielsen MA (2021) Progress and insights toward an effective placental malaria vaccine. *Front Immunol* 12: 634508

Neafsey DE, Juraska M, Bedford T, Benkeser D, Valim C, Griggs A, Lievens M, Abdulla S, Adjei S, Agbenyega T *et al* (2015) Genetic diversity and protective efficacy of the RTS,S/AS01 malaria vaccine. *N Engl J Med* 373: 2025–2037

Nasamu AS, Glushakova S, Russo I, Vaupel B, Oksman A, Kim AS, Fremont DH, Tolia N, Beck JR, Meyers MJ *et al* (2017) Plasmepsins IX and X are essential and druggable mediators of malaria parasite egress and invasion. *Science* 358: 518–522

Pino P, Caldelari R, Mukherjee B, Vahokoski J, Klages N, Maco B, Collins CR, Blackman MJ, Kursula I, Heussler V *et al* (2017) A multistage antimalarial targets the plasmepsins IX and X essential for invasion and egress. *Science* 358: 522–528

Ranson H, Lissenden N (2016) Insecticide resistance in African anopheles mosquitoes: a worsening situation that needs urgent action to maintain malaria control. *Trends Parasitol*. 32: 187–196

Siqueira‐Neto JL, Wicht KJ, Chibale K, Burrows JN, Fidock DA, Winzeler EA (2023) Antimalarial drug discovery: progress and approaches. *Nat Rev Drug Discov* https://doi.org/10.1038/s41573‐023‐00772‐9

Xie SC, Metcalfe RD, Dunn E, Morton CJ, Huang SC, Puhalovich T, Du Y, Wittlin S, Nie S, Luth MR *et al* (2022) Reaction hijacking of tyrosine tRNA synthetase as a new whole‐of‐life‐cycle antimalarial strategy. *Science* 376: 1074–1079

Witkop B (1999) Paul Ehrlich and his magic bullets – revisited. *Proc Am Philos Soc* 143: 540–557

